# Very High Cycle Fatigue Failure Analysis and Life Prediction of Cr-Ni-W Gear Steel Based on Crack Initiation and Growth Behaviors

**DOI:** 10.3390/ma8125459

**Published:** 2015-12-02

**Authors:** Hailong Deng, Wei Li, Tatsuo Sakai, Zhenduo Sun

**Affiliations:** 1 School of Mechanical Engineering, Beijing Institute of Technology, Beijing 100081, China; deng_hl@126.com (H.D.); bdsunzhenduo@126.com (Z.S.); 2 Research Center of Advanced Materials Technology, Ritsumeikan University, Kusatsu 5258577, Shiga, Japan; Sakai@se.ritsumer.ac.jp

**Keywords:** Cr-Ni-W steel, very high cycle fatigue, interior failure, local stress distribution, initiation and growth, life prediction

## Abstract

The unexpected failures of structural materials in very high cycle fatigue (VHCF) regime have been a critical issue in modern engineering design. In this study, the VHCF property of a Cr-Ni-W gear steel was experimentally investigated under axial loading with the stress ratio of *R* = −1, and a life prediction model associated with crack initiation and growth behaviors was proposed. Results show that the Cr-Ni-W gear steel exhibits the constantly decreasing *S-N* property without traditional fatigue limit, and the fatigue strength corresponding to 10^9^ cycles is around 485 MPa. The inclusion-fine granular area (FGA)-fisheye induced failure becomes the main failure mechanism in the VHCF regime, and the local stress around the inclusion play a key role. By using the finite element analysis of representative volume element, the local stress tends to increase with the increase of elastic modulus difference between inclusion and matrix. The predicted crack initiation life occupies the majority of total fatigue life, while the predicted crack growth life is only accounts for a tiny fraction. In view of the good agreement between the predicted and experimental results, the proposed VHCF life prediction model involving crack initiation and growth can be acceptable for inclusion-FGA-fisheye induced failure.

## 1. Introduction

In consideration of the current economics and energy resources, a great number of machinery parts or components, such as axle, wheel, gear, blade, *etc.*, are expected to have a very high cycle fatigue (VHCF) life beyond 10^7^ cycles [1,2]. However, many unexpected failures of structural materials in the VHCF regime are reported one after another [3,4,5], and some peculiar failure mechanisms are not yet well understood. From the viewpoint of safety and reliability, the analysis and assessment methods based on high cycle fatigue can no longer ensure the safety of parts or components in the VHCF regime. To elucidate the VHCF failure mechanisms of structural materials, and then to establish the VHCF damage assessment and life prediction methods, has been more of a concern in the current fatigue field.

The VHCF failure is often found in the experiments of alloy steels as one of the most widely used structural materials [4]. In addition, this failure is often accompanied with a change of failure mechanism from surface to subsurface or interior. The surface failure is mainly induce from surface machining flaws such as scratches or cavities [3], where the interior failure is mainly induced from the metallurgical defects such as non-metallic inclusions [6] or inhomogeneous microstructure [7]. With respect to the interior inclusion-induced failure, a special region can occur in the vicinity of the inclusion if the number of cycle is larger than around 10^6^ cycles. This region was called fine granular area (FGA) by coauthor Sakai [8], and will be used throughout the paper. From the standpoint of dislocation movement and irreversible slip, Sakai *et al.* proposed that the formation processes of FGA can be divided into three stages [9]: (1) Formation of a fine granular layer; (2) Nucleation and coalescence of micro-debondings; and (3) Completed formation of the fine granular area (FGA). Besides that, Shiozawa *et al.* pointed out that the dispersive decohesion of spheroidal carbides from matrix around the inclusion during the long-term cyclic loading is attributed to the formation of FGA [10]. Murakami *et al.* showed that the formation of FGA is caused by the synergistic effect between cyclic stress and hydrogen trapped around the inclusion [11]. Grad *et al.* proposed that the refinement of crystalline structure around the inclusion during cyclic loading leads to fracture surface of FGA [12]. Nakamura *et al.* thought that the cyclic compression between the fine concavo-convex surfaces leads to the formation of FGA around the inclusion [13]. Although the unanimous understanding or conclusion has not yet been drawn, the researchers share a same belief or opinion. That is, the local stress around the inclusion during cyclic loading must be responsible for the formation of FGA, and the FGA governs the VHCF behavior of material.

The $\sqrt{area}$ model associated with material hardness and defect size was developed by Murakami to evaluate the fatigue strength of material with defect-induced failure. Based on this, some methods or models combined with Paris law [14,15,16], FGA size [15,17,18], cumulative damage [19], stress ratio [16,20] and tensile strength [7,19] are proposed to predict fatigue life or strength in the VHCF regime. Although these empirical methods or models are not necessarily appropriate for the experimental results of any steel, they to some extent reflect the failure behaviors of materials under a given condition. However, it is known that the fatigue process consists of crack initiation process and crack growth process. Especially for VHCF occurring at relatively low stress levels, the fatigue life consumed in the crack initiation process should be considered. Unfortunately, studieson the VHCF life prediction associated with the crack initiation and the crack growth are relatively rare.

In the present study, the VHCF property of a gear steel was experimentally investigated under axial loading, and the relevant *S-N* property, failure mechanism and crack size characteristics were analyzed. Based on the finite element modeling, the local stress distribution and the stress gradient effect around the inclusion was addressed, and the relationship between the maximum principal stress and fatigue life was established. Based on the definition of crack initiation and grow processes, a fatigue life model involving both crack initiation and growth was established to evaluate the VHCF life for inclusion-FGA-fisheye induced fatigue failure.

## 2. Experimental Procedure

### 2.1. Material and Specimen

**Figure 1:**
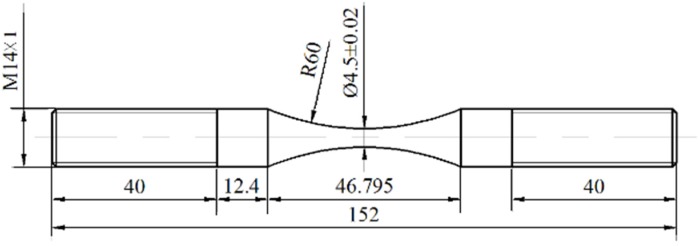
Shape and dimensions of specimen (units: mm).

A Cr-Ni-W gear steel was investigated in this study, and its main chemical composition (wt %) is 0.16 C, 0.19 Si, 0.33 Mn, 1.55 Cr, 4.22 Ni, 0.97 W, 0.01 V, *etc.* The heat treatment processes were described as follows: (a) first quenching: 950 °C × 0.5 h + air cooling; (b) secondary quenching: 850 °C × 0.5 h + air cooling; (c) tempering: 170 °C × 3 h + air cooling. From the annealed steel bar with a diameter of 16 mm, specimens were first machined into the shape of hourglass and then grinded in a direction parallel to the axis of specimen by the grades 600–2000 abrasive paper to final shapes, as shown in Figure 1. The minimum diameter and round notch radius of specimen are 4.5 mm and 60 mm, respectively. Based on stress concentration handbook [21], the stress concentration factor of specimen, *K*_t_ is evaluated to be around 1.02. The tensile strength, yield strength, elastic modulus and Poisson's ratio of Cr-Ni-W gear steel, σ_b_, σ_y_, *E* and ν, are measured to be around 1520 MPa, 1190 MPa, 205 GPa and 0.3, respectively. Furthermore, the hardness distribution along the cross section of specimen is almost uniform and the average value of Vickers hardness (HV) is about 503 kgf/mm^2^.

**Figure 2:**
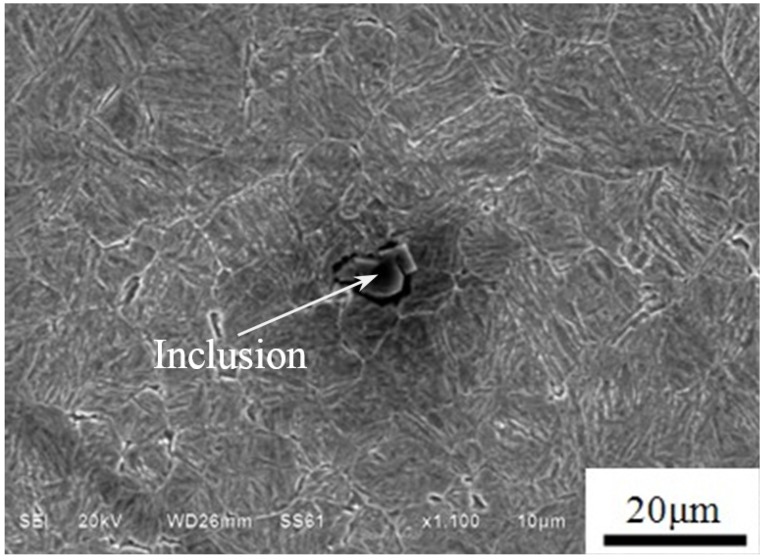
Microstructure and inclusion.

By means of the 4% alcohol nitric acid solution and scanning electronic microscope (SEM), the observed microstructure of heat-treated material is the tempered martensite, as shown in Figure 2. Furthermore, combined with the analysis of energy dispersive X-ray spectrometer (EDS), some non-metallic inclusions of Al_2_O_3_ are found in the steel matrix in Figure 2. Based on the reference [22], the elastic modulus and Poisson's ratio of inclusion, *E_i_* and *υ_i_* are around 390 GPa and 0.25, respectively.

### 2.2. Experimental Method

An axial-type high frequency fatigue testing machine was used to carry out the fatigue test of Cr-Ni-W gear steel, and the testing frequency is around 100 Hz. The fatigue test was performed in an open environment at room temperature, the relevant stress ratio was given −1. During the experiment, the tested specimen has no “self-heat” phenomenon by means of the measurement with an infrared temperature measuring instrument. After the experiment, fracture surfaces of all the failed specimens were carefully observed by SEM, paying attention to the crack initiation sites and failure mechanisms.

## 3. Results and Discussion

### 3.1. S-N Property

**Figure 3:**
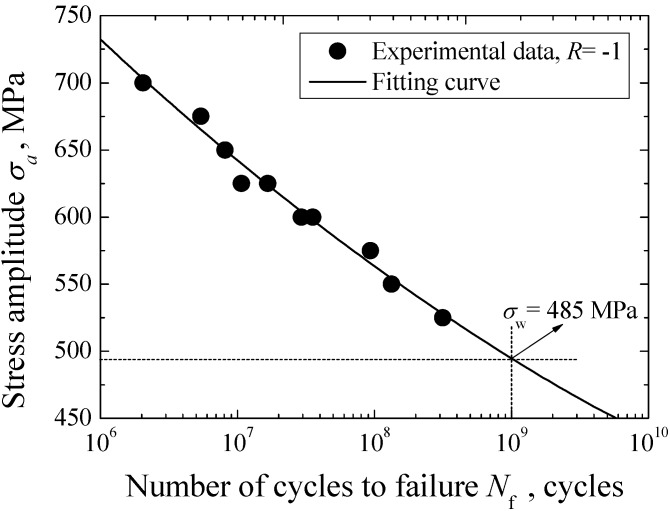
*S-N* curve of Cr-Ni-W alloy steel with interior failure.

The *S-N* data of Cr-Ni-W gear steel under axial loading in the life regime of 10^6^−10^9^ cycles is shown in Figure 3. Based on the preliminary SEM observation of crack initiation site, fatigue failures of specimens are all induced from the subsurface or interior. This means that the interior failure becomes the unique failure mode of Cr-Ni-W gear steel in the VHCF regime. Overall, the Cr-Ni-W gear steel exhibits the constantly decreasing *S-N* property and the traditional fatigue limit corresponding to 10^7^ cycles cannot be observable. In view of the linear distribution characteristics of test data, the Basquin model [23] was used to establish the *S-N* curve of Cr-Ni-W gear steel under axial loading, and a solid line was plotted in Figure 3. Moreover, the stress amplitude corresponding to 10^9^ cycles is approximately defined as the fatigue limit σ_w_ of material. According to the fitted *S-N* curve, the value of σ_w_ is evaluated to be 485 MPa.

### 3.2. Fatigue Failure Mechanism

**Figure 4:**
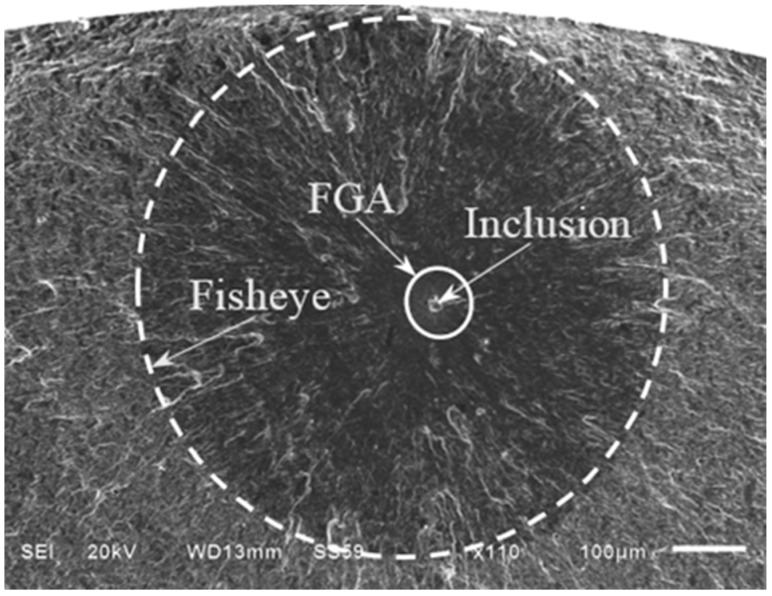
Observation of inclusion, fine granular area (FGA) and fisheye (σ*_a_* = 600 MPa, *N*_f_ = 2.93 × 10^7^ cycles).

Based on the further SEM observation and EDS analysis, the interior failure is all induced by the nonmetallic inclusion contained in material. An approximately circular crack shaped like a fisheye can be observed on the fracture surface, as indicated by a dashed circle in Figure 4. The inclusion is nearly located at the center of the fisheye. It should be noted that the FGA with rougher surface morphology occurs in the vicinity of the inclusion, as indicated by a solid circle in Figure 4. In the region outsider the FGA, furthermore, some radial or “river” patterns can be seen and point to the origin of the crack.

Under cyclic loading, the severe stress concentration inevitably occurs on the interface between the inclusion and the ambient matrix due to the inconsistency of deformation. To a great extent, the inclusion affects the stress distribution on a micro-level and contributes to the interior crack initiation. Moreover, it is undeniable that the local stress distribution around the inclusion play a key role in causing the FGA formation. Furthermore, it should be noted that the crack morphology outside the fisheye is obviously different from that within the fisheye outside the FGA. This is mainly attributed to the different crack growth rate.

### 3.3. Crack Size Characteristics

In view of the approximately circular shape of inclusion, FGA and fisheye, the parameters *R*_inc_*, R*_FGA_ and *R*_Fisheye_ are adapted to indicate the radiuses of inclusion, FGA and fisheye, respectively. By using the graphical software, the measured values of *R*_inc_, *R*_FGA_ and *R*_Fisheye_ are presented in Table 1. It can be seen that the difference for the values of *R*_inc_ is so slight. The average value of *R*_inc_ is evaluated to be 8.7 μm. It can be concluded that the sizes of inclusion are regardless of the applied stress amplitude or fatigue life and dependent on the melting techniques of steel. With the decreasing of applied stress amplitude, *i.e.*, the increasing of fatigue life, the values of FGA and fisheye all tend to increase. That means that the lower stress level is, the sizes of FGA and fisheye are larger.

**Table 1:** Sizes of inclusion, FGA and fisheye.

σ*_a_* (MPa)	*R*_inc_ (μm)	*R*_FGA_ (μm)	*R*_Fisheye_ (μm)
700	10.89	23.34	334.23
675	8.05	24.68	65.86
650	11.85	27.89	229.75
625	8.12	31.45	260.57
625	7.12	30.61	271.83
600	7.86	32.42	366.04
600	10.13	33.49	132.43
575	6.16	36.32	299.69
550	8.56	40.62	421.14
525	8.14	47.48	514.76

### 3.4. Local Stress Distribution around Inclusion

#### 3.4.1. RVE Model

**Figure 5:**
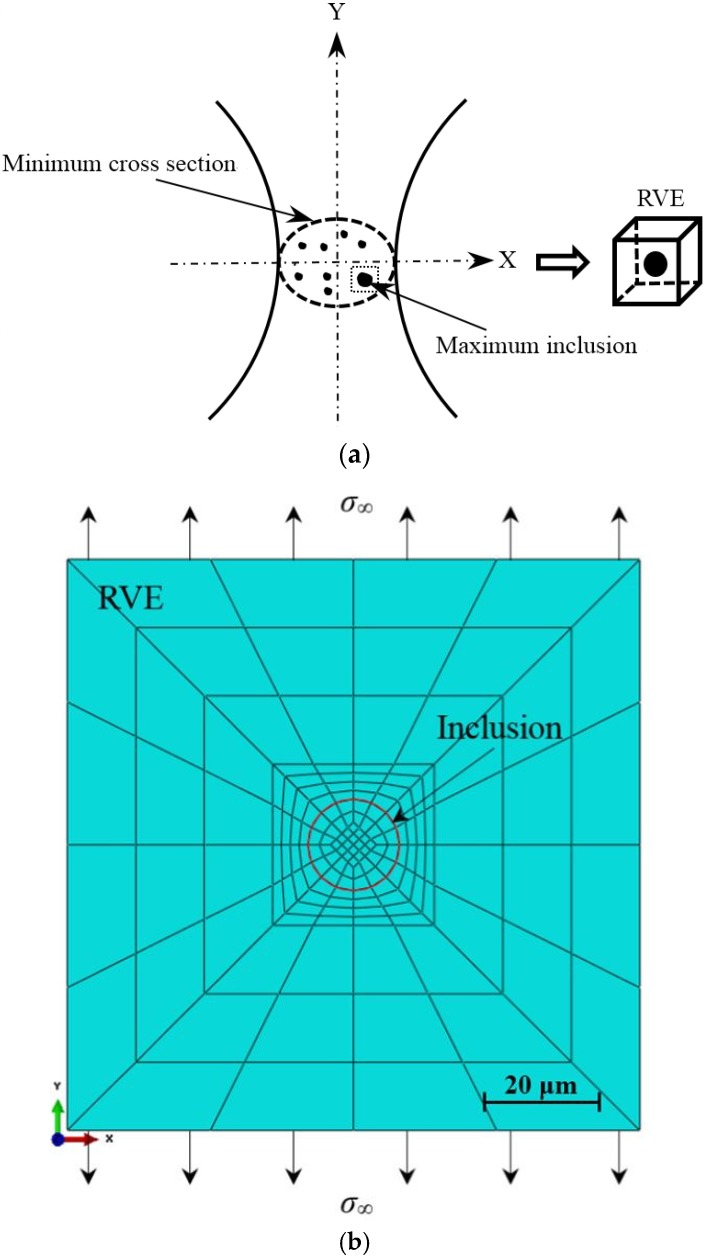
Finite element modeling based on RVE method: (**a**) Definition of RVE; and (**b**) Model and boundary conditions.

As mentioned earlier, the local stress distribution characteristics around the inclusion have a great effect on the VHCF failure of material. To some extent, the local fatigue property around the inclusion governs the overall fatigue property of material in the VHCF regime. Under this circumstance, the representative volume element (RVE) proposed by Hill [24] is used to evaluate the local stress distribution characteristics around the inclusion. The RVE can be considered to be the smallest volume over which a measurement can be made that will yield a value representative of the whole. Based on the SEM observation, the inclusion causing fatigue failure can be viewed as the maximum inclusion on the minimum cross section of specimen under the axial loading, and can be viewed as a sphere. In this study, a cube that contains the maximum inclusion and the adjacent matrix is defined as a RVE, as shown in Figure 5a. During the finite element (FE) modeling of RVE, the type of 4-node-shell elements (CPS4R) as a two-dimension area model was used to simplify the calculation, as shown in Figure 5b. In this figure, the inclusion is defined as the area within the red line and the rest area is the matrix. The side length of RVE is 100 μm, and the radius of inclusion is 8.7 μm which is the average value of *R*_inc_. The RVE which contains the inclusion can be approximately considered as a heterogeneous material. Moreover, considering material mechanical property, the inclusion and the ambient matrix are defined as the different linear elastic materials, and there is no special contact between them. The effect of microstructure such as grain size is not considered in the FE modeling. Furthermore, in the direction of the Y axis, the RVE is subjected to a tensile stress σ_∞_ which is equivalent to the applied normal stress during the experiment.

#### 3.4.2. Effect of Inclusion Properties

**Figure 6:**
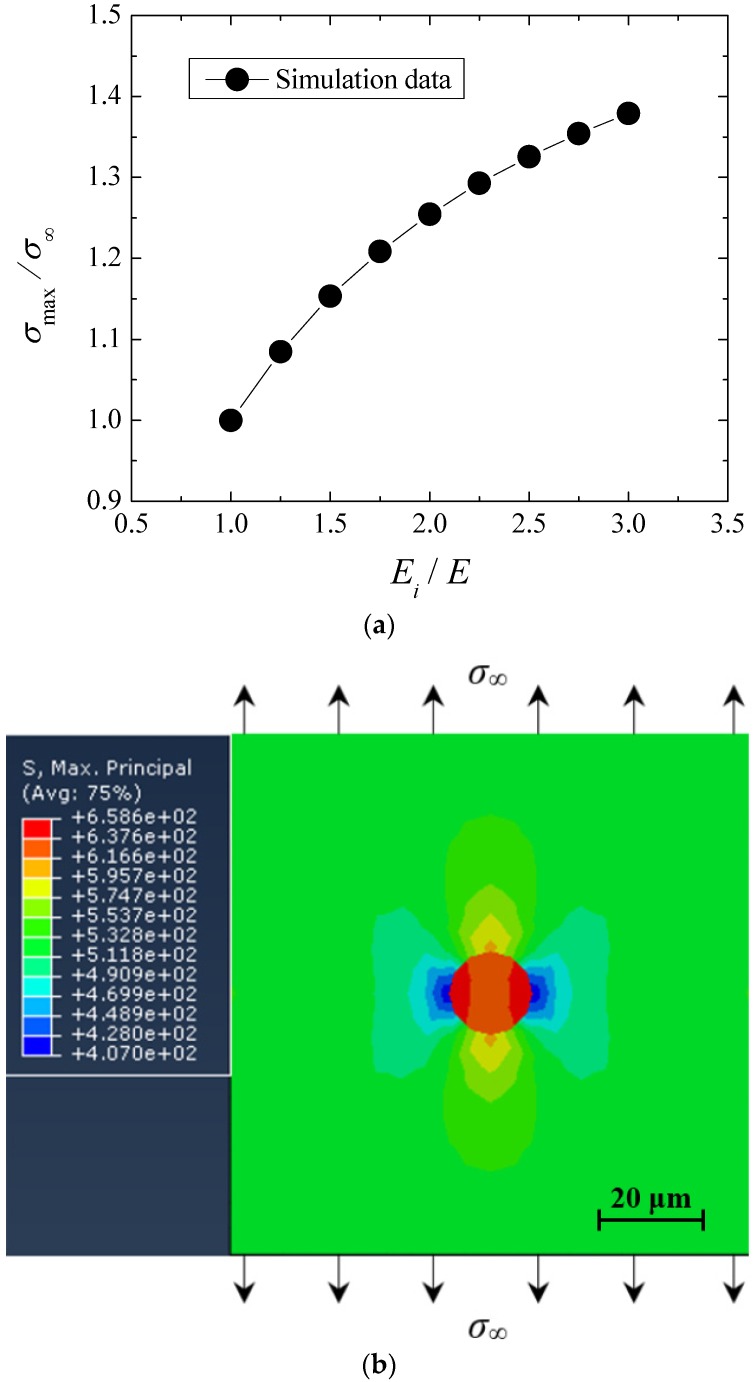
Stress distributions around different inclusions under monotonic tension: (**a**) Relationship between σ_max_/σ_∞_ and *E_i_*/*E*; and (**b**) Stress distribution characteristics for *E_i_* = 420 GPa.

The value of σ_∞_ is set as 525 MPa, the effect of different inclusion properties on the local stress distribution was investigate under monotonic tension. The parameters σ_max_ is used to denote the maximum principal stress around the inclusion. Herein the inclusion property is mainly associated with the change of elastic module. Based on the FE calculation, the relationship between σ_max_/σ_∞_ and *E_i_*/*E* is shown in Figure 6a. In fact, the value of σ_max_/σ_∞_ corresponds to the stress centration factor of inclusion. Obviously, it can be found that the values of σ_max_/σ_∞_ tend to increase with the increase of *E_i_*/*E*. It is known that the elastic module reflects the resistance capacity to elastic deformation. This means that the harder the inclusion is, the maximum principle stress around the inclusion is larger. In other words, the local stress around the inclusion tends to increase with the increase of elastic modulus difference between inclusion and matrix. Corresponding to *E_i_* = 420 GPa, the maximum principal stress distribution diagram is shown in Figure 6b.

#### 3.4.3. Effect of Cyclic Loading

**Figure 7:**
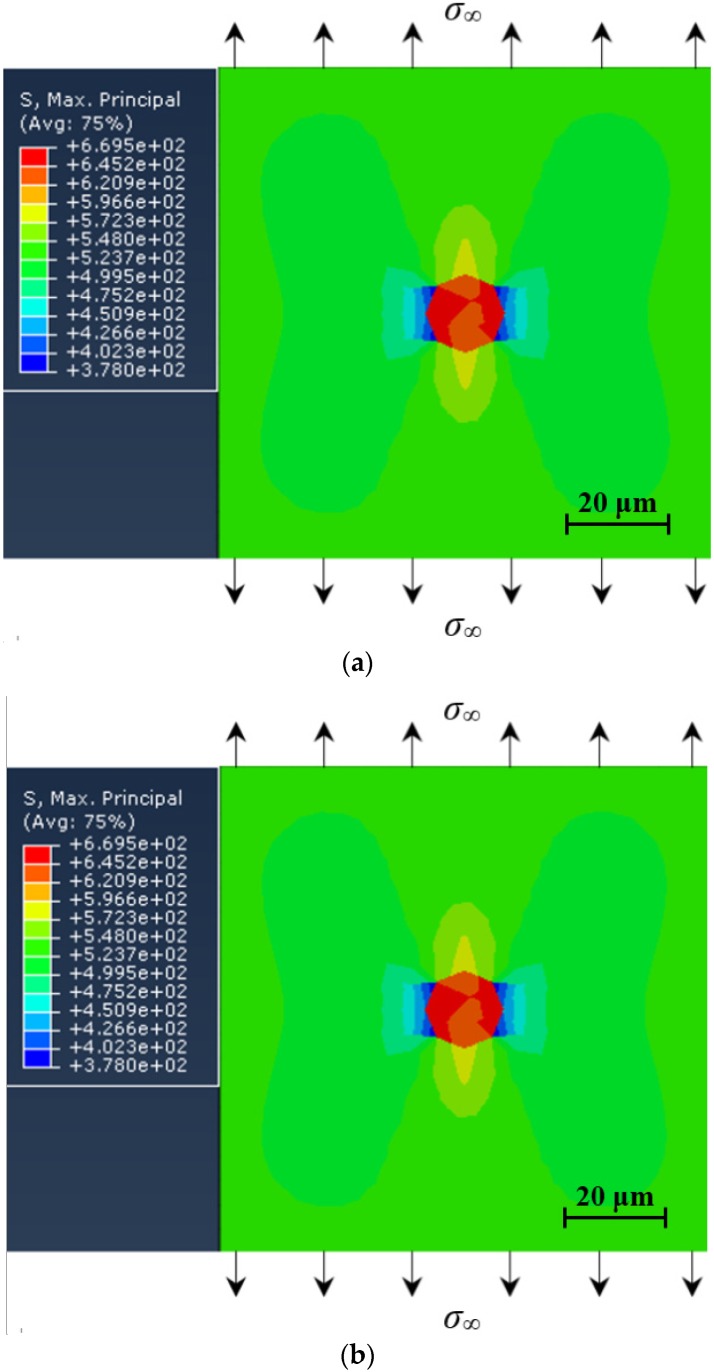
Stress distribution around inclusion under cyclic loading: (**a**) at *N* = 10 cycles; and (**b**) at *N* = 10^3^ cycles.

Under the condition of σ_∞_ = 525 MPa and *E_i_* = 420 GPa, the effect of cyclic loading on the local stress distribution around the inclusion was investigated. It should be noted that considering the likelihood of cyclic hardening of material under cyclic loading, a combined isotropic-kinematic hardening constitutive relation was accounted for the FE-modeling under cyclic loading. Conversely, only the linear-elastic constitutive relation was defined under static loading. The obtained maximum principal stress distribution diagrams under cyclic loading of 10 and 10^3^ cycles are shown in Figure 7. Compared with the result in Figure 6b, first, it can be concluded that cyclic loading has a little effect on the local stress distribution around the inclusion. Then, based on the comparison between results in Figure 7a,b, the number of loading cycles has no significant effect on the local stress distribution around the inclusion.

#### 3.4.4. Maximum Principal Stress Life Curve

**Figure 8:**
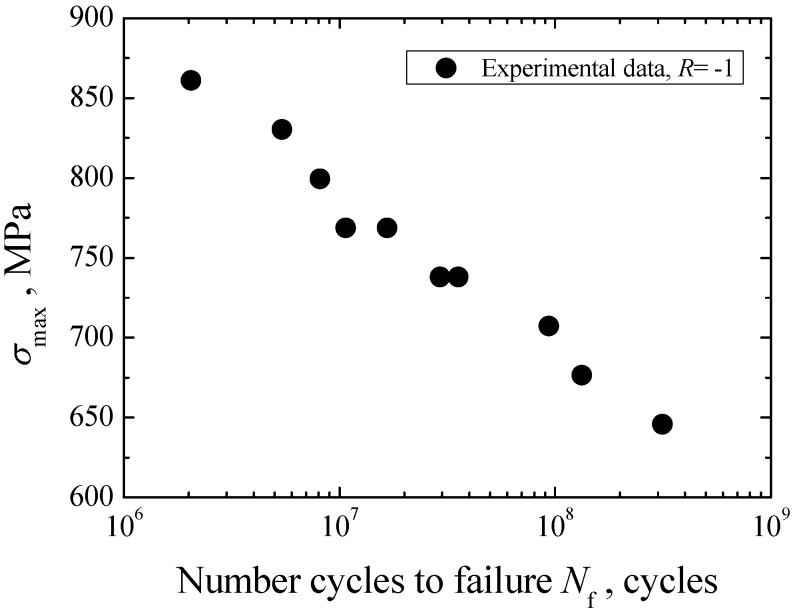
Evaluation of maximum principal stress at the interface between inclusion and matrix.

**Figure 9:**
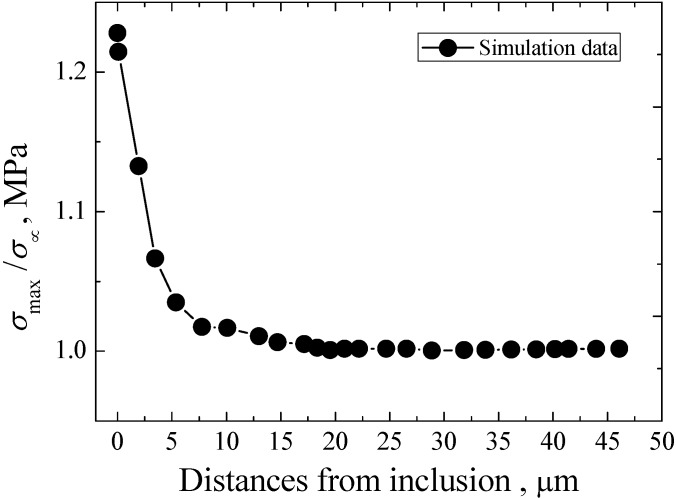
Establishment of maximum principal stress gradient curve.

Based on the applied stress amplitudes and the sizes of inclusions in Table 1, the relevant maximum principle stresses around different inclusions at different stresses can be obtained by using the RVE model. The relationship between the obtained maximum principal stresses and the fatigue life can be established, as shown in Figure 8. Similarly, the maximum principal stress has a tendency to increase with the decrease of fatigue life. On the other hand, corresponding to the inclusion size of 8.14 μm and the stress amplitude of 525 MPa, the ratio of σ_max_/σ_∞_ as a function of the distance from the edge of inclusion is shown in Figure 9. It can be found that with the increase of distance from the edge of inclusion, the value of σ_max_/σ_∞_ tends to decrease. About at the distance of 20 μm, the value of σ_max_/σ_∞_ begins to approach 1. Approximately, it can be concluded that the higher stress concentration and stress gradient effect around the inclusion are greatly related to the formation of FGA.

### 3.5. Crack Initiation Life

#### 3.5.1. Local Stress-Life Model

**Figure 10:**
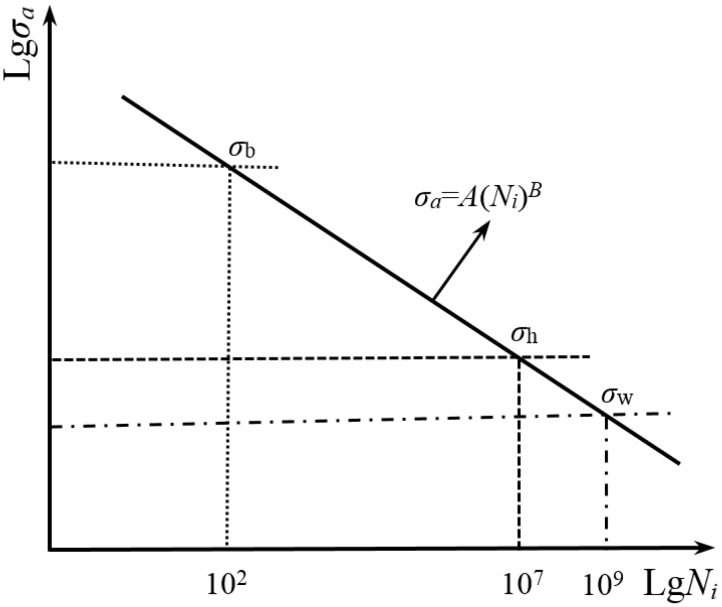
Basquin *S*-*N* relationship in lg-lg scale.

The local stress-life curve can be used to predict the crack initiation life, *N_i_* [25]. Based on Basquin equation [23], a crack initiation life model limited in the life regime of 10^2^–10^7^ cycles was proposed and expressed as [26]

(1) $$N_{i}=10^{7}{\left(\frac{700}{\sigma_{a}}\right)}^{\frac{4}{\log_{10}\sigma_{\text{b}}-2.85}}$$

where the tensile strength σ_b_ is related to the local hardness, about σ_b_ = 3.2 HV, and 700 MPa is assumed to be the fatigue limit value corresponding to fatigue life of 10^7^ cycles. The relevant schematic diagram is shown in Figure 10, where σ_b_ also approximately indicates the fatigue strength at fatigue life of 10^2^ cycles and the parameter σ_h_ denotes the fatigue strength at fatigue life of 10^7^ cycles, *i.e.*, σ_h_ = 700 MPa. For the tested gear steel in this study, the evaluated value of σ_b_ is about 1609 MPa based on σ_b_ = 3.2 HV, somewhat larger than the experimental value of 1520 MPa. The difference between them is slight. For the VHCF failure, however, the definition of fatigue limit corresponding to fatigue life of 10^7^ cycles is apparently not suitable. Therefore, the fatigue strength σ_w_ corresponding to the fatigue life of 10^9^ cycles is used to replace σ_h_. Based on the Basquin equation σ*_a_* = *A*(*N*)*^B^*, the value of σ_w_ can be given by

(2) $$\sigma_{\text{w}}=A{\left(10^{9}\right)}^{B}$$

where parameters *A* and *B* are coefficient and exponent, respectively. Combined with Equation (2), the values of *A* and *B* can be respectively expressed as

(3) $$A=\frac{\sigma_{\text{w}}}{10^{9B}}$$

(4) $$B=\frac{\log_{10}\sigma_{\text{w}}-\log_{10}\sigma_{\text{b}}}{7}$$

Finally, Combined with the evaluated local maximum stress, the crack initiation life model based on the local stress-life at 10^9^ cycles can be established as

(5) $$N_{i}=10^{9}{\left(\frac{\sigma_{\text{w}}}{\sigma_{\text{max}}}\right)}^{\frac{7}{\log_{10}\sigma_{\text{b}}-\log\sigma_{\text{w}}}}$$

#### 3.5.2. Tanaka-Mura Model

Based on dislocation-energy method, a crack initiation life model associated with the inclusion de-bonding or cracking was proposed by Tanaka-Mura and expressed as [27]

(6) $$N_{i}=\left[\frac{\mu_{\text{m}}\left(\mu_{\text{m}}+\mu_{\text{inc}}\right)}{\mu_{\text{inc}}}\right]{\left(\frac{h}{h+l}\right)}^{2}\frac{4W_{\text{s}}}{{\left(\Delta \tau -2\tau_{\text{f}}\right)}^{2}}\frac{1}{R_{\text{inc}}}$$

where μ_m_ is the shear modulus of matrix, μ_inc_ is the shear modulus of inclusion, *h* and *l* are semi-minor and semi-major of the slip band zone respectively, *W*_s_ is the specific fracture energy for a unit area along the slip band, Δτ is the local shear stress range and τ_f_ is the critical shear stress. Generally, the value of *l* is defined as the half of grain size. The relationship between *E* and shear modulus μ is given by:

(7) μ=E/(2+2υ)

Furthermore, based on the maximum octahedral shear stress criterion, the relationship between the critical octahedral shear stress, τ_oct_ and the yield strength of material, σ_y_ can be defined as [20]:

(8) $$\tau_{\text{oct}}=\sqrt{2}\sigma_{\text{y}}/3$$

Accordingly, the values of Δτ and τ_f_ in Equation (6) can be expressed as:

(9) $$\Delta \tau =\sqrt{2}\Delta \sigma /3\text{ and }\tau_{f}=\sqrt{2}\sigma_{\text{w}}/3$$

where σ_w_ is the critical stress below which crack does not initiate, and herein defined as the fatigue strength corresponding to fatigue life of 10^9^ cycles. Combined with Equations (6) and (9), the crack initiation model with the FGA formation is obtained as:

(10) $$N_{i}=\left[\frac{\mu_{\text{m}}\left(\mu_{\text{m}}+\mu_{\text{inc}}\right)}{\mu_{\text{inc}}}\right]{\left(\frac{h}{h+l}\right)}^{2}\frac{9W_{\text{s}}}{2{\left(\sigma_{\max}-\sigma_{\text{w}}\right)}^{2}}\frac{1}{R_{\text{inc}}}$$

When *h* is much less than *l*, the Equation (6) can become

(11) $$N_{i}=\left[\frac{\mu_{\text{m}}\left(\mu_{\text{m}}+\mu_{\text{inc}}\right)}{\mu_{\text{inc}}}\right]{\left(\frac{h}{l}\right)}^{2}\frac{9W_{\text{s}}}{2{\left(\sigma_{\max}-\sigma_{\text{w}}\right)}^{2}}\frac{1}{R_{\text{inc}}}$$

If the slip band zone is circular, *i.e., h* is the same as *l*, the Equation (10) can be simplified as

(12) $$N_{i}=\left[\frac{\mu_{\text{m}}\left(\mu_{\text{m}}+\mu_{\text{inc}}\right)}{\mu_{\text{inc}}}\right]\frac{9W_{\text{s}}}{8{\left(\sigma_{\max}-\sigma_{\text{w}}\right)}^{2}}\frac{1}{R_{\text{inc}}}$$

For the fracture energy *W*_s_, moreover, it can be given by [28]

(13) $$W_{\text{s}}=\Delta K_{\text{th}}^{2}/\left(2E\right)$$

#### 3.5.3. Modified Chan Model 

The Tanaka-Mura model is based on the assumption that all the dislocations in the slip band zone contribute to the crack initiation. However, studies show that not all dislocations contribute to the crack initiation, and the number of dislocations that contribute to the crack initiation is greatly related to the crack size [29]. In a slip band zone, the effective number of dislocations causing crack initiation, *n*_c_, can be given by [30]

(14) $$n_{\text{c}}=0.05\left(\frac{2l}{bh}\right)\sqrt{\frac{cW_{\text{eq}}}{2l\mu_{\text{m}}}}$$

where *b* is the magnitude of the Burgers vector; *W*_eq_ is the strain energy stored in the dislocations of an equivalent slipband and *c* is the crack length. From incipient crack initiation, *W*_eq_ can be expressed as

(15) $$W_{\text{eq}}=4l\gamma_{\text{S}}$$

where γ_s_ is the surface energy of the crack. In this case, the value of *c* is given by

(16) $$c=n_{\text{c}}b$$

Combined with Equation (14), yield

(17) $$c=0.005{\left(\frac{2l}{h}\right)}^{2}\frac{\gamma_{\text{s}}}{\mu_{\text{m}}}$$

Combined with Equation (10) and imposing the condition that γ_s_ = *W*_s_ [29], leads to

(18) $$N_{i}=\left[\frac{\mu_{\text{m}}\left(\mu_{\text{m}}+\mu_{\text{inc}}\right)}{0.005\mu_{\text{inc}}}\right]\frac{h^{4}}{l^{2}{\left(h+l\right)}^{2}}\frac{9\mu_{\text{m}}}{8{\left(\sigma_{\max}-\sigma_{\text{w}}\right)}^{2}}\frac{c}{R_{\text{inc}}}$$

Taking the square root of Equation (18), yield

(19) $$N_{i}^{1/2}={\left[\frac{\mu_{\text{m}}\left(\mu_{\text{m}}+\mu_{\text{inc}}\right)}{0.005\mu_{\text{inc}}}\right]}^{1/2}\frac{h^{2}}{l\left(h+l\right)}\frac{3\sqrt{2}\mu_{\text{m}}^{\text{1/2}}}{4(\sigma_{\max}-\sigma_{\text{w}})}{\left(\frac{c}{R_{\text{inc}}}\right)}^{1/2}$$

For the better agreement with the experimental data, the exponent of *N_i_* can be generalized to a variable α (0 < α < 1) [31]. Therefore, Equation (19) can be rewritten as

(20) $$N_{i}^{\alpha }={\left[\frac{\mu_{\text{m}}\left(\mu_{\text{m}}+\mu_{\text{inc}}\right)}{0.005\mu_{\text{inc}}}\right]}^{1/2}\frac{h^{2}}{l\left(h+l\right)}\frac{3\sqrt{2}\mu_{\text{m}}^{\text{1/2}}}{4(\sigma_{\max}-\sigma_{\text{w}})}{\left(\frac{c}{R_{\text{inc}}}\right)}^{1/2}$$

In this study, it is assumed that the elliptic slip band zone is expanded to the whole FGA. Thus, Equation (20) can be rewritten

(21) $$N_{i}^{\alpha }={\left[\frac{\mu_{\text{m}}\left(\mu_{\text{m}}+\mu_{\text{inc}}\right)}{0.005\mu_{\text{inc}}}\right]}^{1/2}\frac{h_{\text{FGA}}^{2}}{l_{\text{FGA}}\left(h_{\text{FGA}}+l_{\text{FGA}}\right)}\frac{3\sqrt{2}\mu_{\text{m}}^{\text{1/2}}}{4(\sigma_{\max}-\sigma_{\text{w}})}{\left(\frac{c}{R_{\text{inc}}}\right)}^{1/2}$$

where *h*_FGA_ and *l*_FGA_ are the semi-minor and semi-major of FGA. Herein, *l*_FGA_ is assumed to be equivalent to *R*_FGA_ and *h*_FGA_ is a fitting parameter just like α. Based on the established maximum stress-life data and the measured sizes of inclusion and FGA, the values of α and *h*_FGA_ can be determined. The crack initiation life corresponding to different *c*-values can be obtained as

(22) $$N_{i}={\left[\frac{\mu_{\text{m}}\left(\mu_{\text{m}}+\mu_{\text{inc}}\right)}{0.005\mu_{\text{inc}}}\right]}^{1/2\alpha }{\left(\frac{h_{\text{FGA}}^{2}}{l_{\text{FGA}}\left(h_{\text{FGA}}+l_{\text{FGA}}\right)}\frac{3\sqrt{2}\mu_{\text{m}}^{\text{1/2}}}{4(\sigma_{\max}-\sigma_{\text{w}})}\right)}^{1/\alpha }{\left(\frac{c}{R_{\text{inc}}}\right)}^{1/2\alpha }$$

#### 3.5.4. Comparison of Model

**Figure 11:**
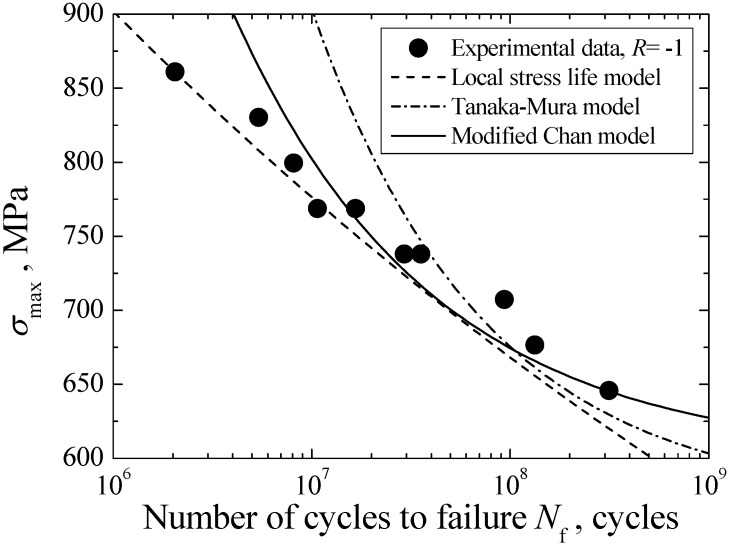
Comparison of three crack initiation life models.

According to Equations (5), (12) and (22), the crack initiation life curves corresponding to these three models are plotted in Figure 11, where the curve established by using modified Chan model corresponds to the crack length of FGA size, *i.e.*, *c* = *R*_FGA_ − *R*_inc_. The established crack initiation life curves all exhibit the continuously descending trend, which is consistent with the variation trend of experimental data. From the statistical consideration, the fitting correlation coefficents associated with local stress-life model, Tanaka-Mura model and Modified Chan model are evaluated to be 0.977, 0.958 and 0.992, respectively. Obviously, the predicted result based on modified Chan model shows the better agreement between the predicted and experimental data. Moreover, all predicted results reveal that the crack initiation life accounts for the vast majority of the total fatigue life.

**Figure 12:**
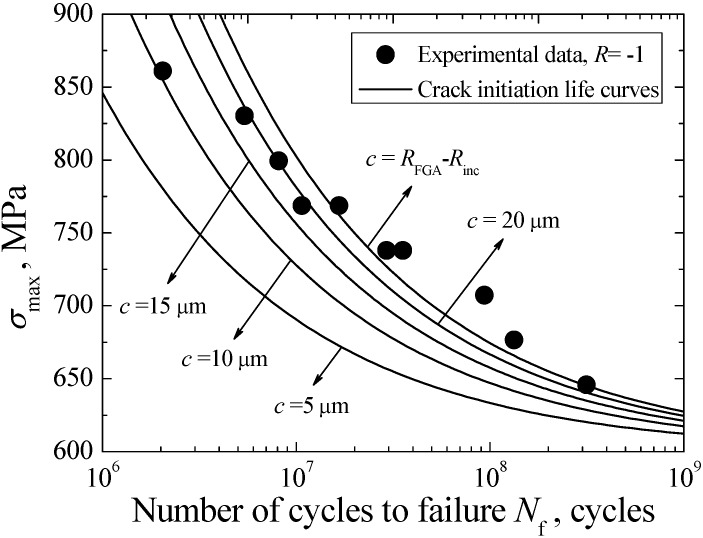
Predicted crack initiation lives by using modified Chan model at different *c*-values.

Based on a modified Chan model, the crack initiation life curves corresponding to different *c*-values can be established, as shown in Figure 12. It can be seen from the figure that the crack initiation life tends to increase with the increase of crack size at a certain stress. Furthermore, the need crack initiation life to induce a certain crack size is longer at low stress than at high stress. In view of the fact that the predicted crack nucleation lives associated with the FGA sizes are in good agreement with the experimental data, it reveals that the VHCF life is mainly consumed in the crack initiation stage inside the FGA.

### 3.6. Crack Growth Life

**Figure 13:**
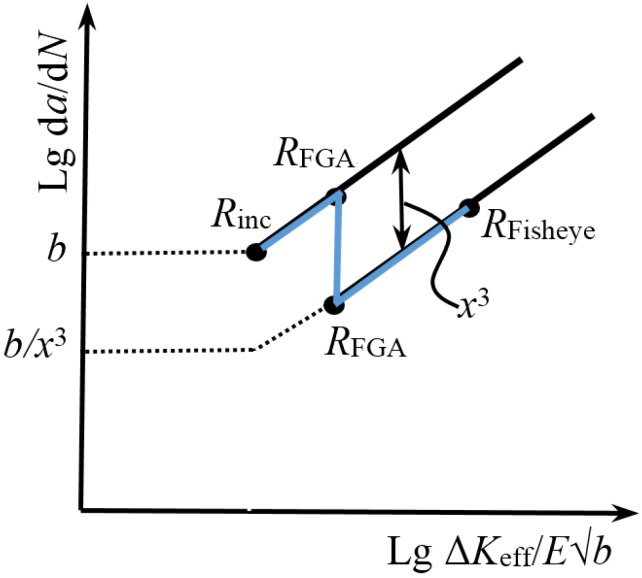
Schematic of small and long crack growth behaviors.

Combined with the previous studies [9], the crack growth for the interior inclusion-FGA-fisheye failure in the VHCF regime can be divided into two stages: (i) small crack growth from inclusion to FGA; and (ii) long crack growth from FGA to fisheye, as schematically shown in Figure 13. A crack growth rate equation was used to describe the crack growth behaviors in these two stages, and expressed as [32]

(23) $$\frac{da}{dN}=b{\left(\frac{\Delta K_{\text{eff}}}{E\sqrt{b}}\right)}^{3}$$

where Δ*K*_eff_ is the effective stress intensity factor range at a crack tip. For a circular crack with a radius *a*, the value of Δ*K*_eff_ can be defined as [33]

(24) $$\Delta K_{\text{eff}}=\frac{2}{\pi }\Delta \sigma \sqrt{\pi a}$$

For the inclusion, hence, its effective stress intensity factor range, Δ*K*_inc_ can be expressed as

(25) $$\Delta K_{\text{inc}}=\frac{2}{\pi }\Delta \sigma_{\max}\sqrt{\pi R_{\text{inc}}}$$

In combination with Equations (23) and (25), the crack growth rate for stage (i) can be given by

(26) $$\frac{da}{dN}=b{\left(\frac{2\Delta \sigma_{\max}\sqrt{\pi a}}{\pi E\sqrt{b}}\right)}^{3}=b{\left(\frac{\Delta K_{\text{inc}}}{E\sqrt{b}}\right)}^{3}{\left(\frac{a}{R_{\text{inc}}}\right)}^{3/2}=b{\left(\frac{a}{R_{\text{inc}}}\right)}^{3/2}$$

where $\Delta K_{\text{inc}}/E\sqrt{b}=1$ is the threshold corner location with *da*/*dN* = *b*. That is,

(27) $$1=\frac{\Delta K_{\text{inc}}}{E\sqrt{b}}=\frac{2\Delta \sigma_{\max}\sqrt{\pi R_{\text{inc}}}}{\pi E\sqrt{b}}\;\;$$

Integrating Equation (26) from *R*_inc_ to *R*_FGA_, the crack growth life at stage (i), *N*_1_, is obtained as

(28) $$N_{1}=\frac{\pi E^{2}}{2{\left(\Delta \sigma_{\max}\right)}^{2}}\left[1-\sqrt{\frac{R_{\text{inc}}}{R_{\text{FGA}}}}\right]$$

In view of the difference between the small and long crack growth behaviors, the growth rate in stage (ii) is reduced by the fact “1/*x*^3^” in comparison with the growth rate in stage (i). Thus, the crack growth rate for stage (ii) is given as

(29) $$\frac{da}{dN}=\frac{b}{x^{3}}{\left(\frac{2\Delta \sigma_{\max}\sqrt{\pi a}}{\pi E\sqrt{b}}\right)}^{3}=\frac{b}{27}{\left(\frac{a}{R_{\text{inc}}}\right)}^{3/2}$$

Integrating Equation (29) from *R*_FGA_ to *R*_Fisheye_, the crack growth life at stage (ii), *N*_2_, is obtained as

(30) $$N_{2}=\frac{27\pi E^{2}}{2{\left(\Delta \sigma_{\max}\right)}^{2}}\left[\sqrt{\frac{R_{\text{inc}}}{R_{\text{FGA}}}}-\sqrt{\frac{R_{\text{inc}}}{R_{\text{Fisheye}}}}\right]$$

Combined with Equations (28) and (30), the total crack growth life, *N*_p_ can be expressed as

(31) $$N_{\text{p}}=N_{1}+N_{2}\;=\frac{\pi E^{2}}{2{\left(\Delta \sigma_{\max}\right)}^{2}}\left[1+26\sqrt{\frac{R_{\text{inc}}}{R_{\text{FGA}}}}-27\sqrt{\frac{R_{\text{inc}}}{R_{\text{Fisheye}}}}\right]$$

**Figure 14:**
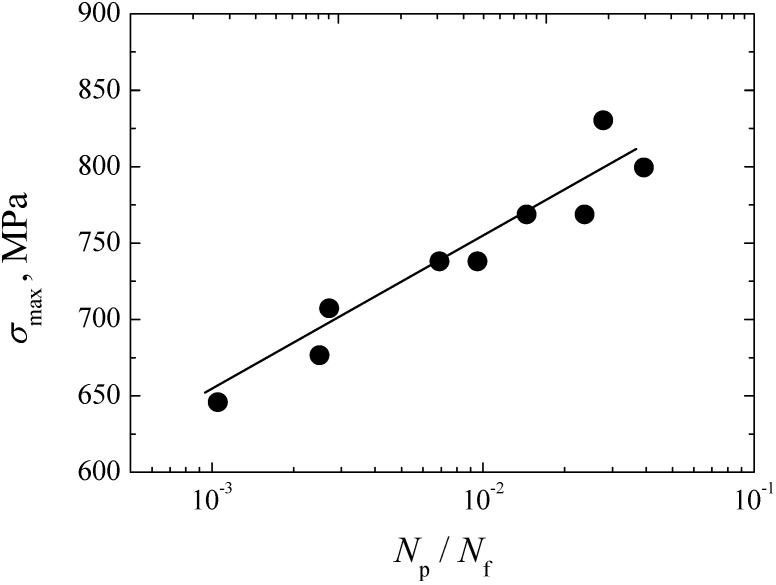
Relationship between σ_max_ and *N*_p_/*N*_f_.

The predicted crack growth lives are presented in Table 2, where the total crack growth lives are only scattered in the range of 1 × 10^5^–4 × 10^5^ cycles. With the decrease of stress amplitude, the predicted crack growth life has an increase tendency. In contrast, the life for the small crack growth from inclusion to FGA is shorter than that for the long crack growth from FGA to fisheye. This means that the crack growth life is mainly consumed in the long crack growth stage. On the other hand, the ratio of the predicted crack growth life to the experimental life, *N*_p_/*N*_f_ considered as a function of σ_max_, is shown in Figure 14. It can be found that with the increase of stress, the proportion of the crack growth life in the total fatigue life tends to increase. At a relatively low stress level, the crack growth life only accounts for a very tiny fraction of the total fatigue life. Conversely, it reveals that the VHCF life is mostly consumed in the crack initiation stage.

**Table 2:** Predicted results of crack propagation life.

σ*_a_* (MPa)	σ_max_ (MPa)	*N*_1_ (cycles)	*N*_2_ (cycles)	*N*_p_ (cycles)
700	842	7.36 × 10^3^	3.15 × 10^5^	3.23 × 10^5^
675	837	1.01 × 10^4^	1.40 × 10^5^	1.51 × 10^5^
650	781	9.40 × 10^3^	3.10 × 10^5^	3.19 × 10^5^
625	783	1.32 × 10^4^	2.40 × 10^5^	2.54 × 10^5^
625	792	1.36 × 10^4^	2.27 × 10^5^	2.41 × 10^5^
600	761	1.44 × 10^4^	2.65 × 10^5^	2.80 × 10^5^
600	725	1.41 × 10^4^	2.31 × 10^5^	2.45 × 10^5^
575	714	1.90 × 10^4^	2.34 × 10^5^	2.53 × 10^5^
550	672	1.97 × 10^4^	3.11 × 10^5^	3.31 × 10^5^
525	645	2.33 × 10^4^	3.08 × 10^5^	3.31 × 10^5^

### 3.7. Total Fatigue Life Model

**Figure 15:**
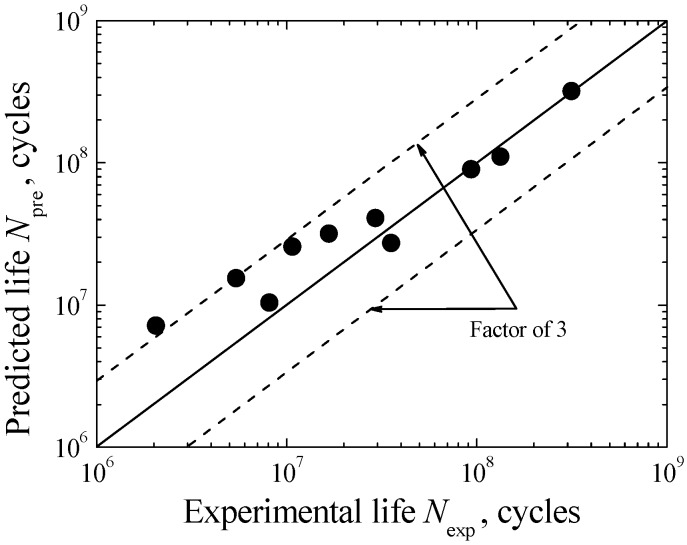
Comparison of predicted and experimental lives.

Based on Equations (22) and (31), the VHCF life prediction model associated with the inclusion-FGA-fisheye failure can be obtained as

(32) $$\begin{array}{l} N_{\text{pre}}=N_{i}+N_{\text{p}}\\ \;\;\;\;\;={\left[\frac{\mu_{\text{m}}\left(\mu_{\text{m}}+\mu_{\text{inc}}\right)}{0.005\mu_{\text{inc}}}\right]}^{1/2\alpha }{\left(\frac{h_{\text{FGA}}^{2}}{l_{\text{FGA}}\left(h_{\text{FGA}}+l_{\text{FGA}}\right)}\frac{3\sqrt{2}\mu_{\text{m}}^{\text{1/2}}}{4(\sigma_{\max}-\sigma_{\text{w}})}\right)}^{1/\alpha }{\left(\frac{c}{R_{\text{inc}}}\right)}^{1/2\alpha }\\ \;\;\;\;\;\;\;\;\;+\frac{\pi E^{2}}{2{\left(\Delta \sigma_{\max}\right)}^{2}}\left[1+26\sqrt{\frac{R_{\text{inc}}}{R_{\text{FGA}}}}-27\sqrt{\frac{R_{\text{inc}}}{R_{\text{Fisheye}}}}\right] \end{array}$$

where *N*_pre_ is the predicted total fatigue life. According to the comparison between the predicted and experimental lives shown in Figure 15, the agreement is fairly good within the factor-of-three boundaries. On the whole, the predicted result is satisfactory and the above-established model is acceptable. Furthermore, it should be noted that herein proposed VHCF life prediction model involves the crack initiation and growth processes, and reflects the inclusion-FGA-fisheye induced failure mechanism. As long as the material exhibits this failure mechanism, the proposed model can be used well. However, from the viewpoint of reliability, this model is still needed to verify by more experimental data of diverse materials.

## 4. Conclusions

Main conclusions obtained in this study are summarized as follows:

(1) The Cr-Ni-W gear steel exhibits the constantly decreasing *S-N* property without traditional fatigue limit, and the fatigue strength corresponding to 10^9^ cycles is around 485 MPa.
(2) The inclusion-FGA-fisheye induced failure becomes the main failure mechanism of Cr-Ni-W gear steel in the VHCF regime.
(3) By using the finite element analysis of representative volume element, the local stress around the inclusion tends to increase with the increase of elastic modulus difference between inclusion and matrix.
(4) The local stress-life model, Tanaka-Mura model and modified Chan model were used to evaluate the crack initiation life, and the predicted results based on modified Chan model are better.
(5) The predicted crack initiation life occupies the most majority of total fatigue life, while the predicted crack growth life is only accounts for a tiny fraction.
(6) The established VHCF life prediction model involving crack initiation and growth for inclusion-FGA-fisheye induced failure is acceptable based on the good agreement between the predicted and experimental results.
